# Prognostic Value of Microvascular Invasion in Eight Existing Staging Systems for Hepatocellular Carcinoma: A Bi-Centeric Retrospective Cohort Study

**DOI:** 10.3389/fonc.2021.726569

**Published:** 2021-12-16

**Authors:** Yan-Jun Xiang, Kang Wang, Yi-Tao Zheng, Hong-Ming Yu, Yu-Qiang Cheng, Wei-Jun Wang, Yun-Feng Shan, Shu-Qun Cheng

**Affiliations:** ^1^ Department of Hepatobiliary Surgery, The First Affiliated Hospital, Wenzhou Medical University, Wenzhou, China; ^2^ Department of Hepatic Surgery VI, Eastern Hepatobiliary Surgery Hospital, Second Military Medical University, Shanghai, China

**Keywords:** microvascular invasion, hepatocellular carcinoma, staging system, prognosis, bi-centeric

## Abstract

**Background:**

Microvascular invasion (MVI) is a significant risk factor affecting survival outcomes of patients after R0 liver resection (LR) for hepatocellular carcinoma (HCC). However, whether the existing staging systems of hepatocellular carcinoma can distinguish the prognosis of patients with MVI and the prognostic value of MVI in different subtypes of hepatocellular carcinoma remains to be clarified.

**Methods:**

A dual-center retrospective data set of 1,198 HCC patients who underwent R0 LR was included in the study between 2014 and 2016. Baseline characteristics and staging information were collected. Homogeneity and modified Akaike information criterion (AICc) were compared between each system. And the prognostic significance of MVI for overall survival (OS) was studied in each subgroup.

**Results:**

In the entire cohort, there were no significant survival differences between Cancer of the Liver Italian Program (CLIP) score 2 and 3 (p = 0.441), and between Taipei Integrated Scoring System (TIS) score 3 and 4 (p = 0.135). In the MVI cohort, there were no significant survival differences between Barcelona Clinic Liver Cancer stages B and C (p=0.161), CLIP scores 2 and 3 (p = 0.083), TIS scores 0 and 1 (p = 0.227), TIS scores 2 and 3 (p =0.794), Tokyo scores 3 and 4 (p=0.353), and American Joint Committee on Cancer Tumor-Node-Metastasis 7^th^ stage I and II (p=0.151). Among the eight commonly used HCC staging systems, the Hong Kong Liver Cancer (HKLC) staging system showed the highest homogeneity and the lowest AICc value in both the entire cohort and MVI cohort. In each subgroup of the staging systems, MVI generally exhibited poor survival outcomes.

**Conclusions:**

The HKLC staging system was the most accurate model for discriminating the prognosis of MVI patients, among the eight staging systems. Meanwhile, our findings suggest that MVI may be needed to be incorporated into the current HCC staging systems as one of the grading criteria.

## Introduction

Hepatocellular carcinoma (HCC) is the leading cause of cancer-related death and responsible for more than 700,000 deaths annually (1). Liver resection (LR) or liver transplantation remains the first-line treatment method for patients with early or intermediate stage of HCC (2–4). Unfortunately, the 5-year recurrence rate is as high as 70–80% after curative liver resection, which severely limits the long-term survival of patients with HCC (5, 6).

Microvascular invasion (MVI), defined as “a cancer cell nest with >50 cells in the endothelial vascular lumen under microscopy” (7), is considered an early means of cancer cell spread through the vasculature and a key factor affecting the recurrence and long-term survival of patients with HCC (8–12). However, some authors have recently suggested that MVI is not a prognostic factor for all HCC patients. The long-term survival of small HCC (≤2 cm) is excellent and not influenced by MVI (13), and the clinical value of MVI in patients at Barcelona Clinic Liver Cancer (BCLC) stages 0 or B is limited (12, 14). Thus, the prognostic significance of MVI in various HCC staging systems still needs further investigation.

To date, several staging systems have been proposed to stratify HCC patients into different subgroups for better treatment decision-making and prognostic prediction (15). Among these, the BCLC staging system is recommended by the European Association for the Study of the Liver (EASL) and the American Association for the Study of Liver Diseases (AASLD) (2, 3). Compared with the BCLC system, the recently proposed Hong Kong Liver Cancer (HKLC) staging system provides better prognostic ability and a more aggressive treatment algorithm (16). In addition to the BCLC and HKLC systems, multiple staging systems have been proposed, including Cancer of the Liver Italian Program (CLIP), Taipei Integrated Scoring System (TIS), Tumor-Node-Metastasis (TNM) by Liver Cancer Study Group of Japan (LCSGJ), Tokyo Score, American Joint Cancer Committee (AJCC) TNM 7^th^ edition, and Okuda staging system (17–21). Pursuing an optimal staging system for HCC has generated a gradually upward interest over the past two decades, and this lack of consensus may stem from the heterogeneity of the underlying liver diseases and different preferences for treatment modalities worldwide (22, 23). A recent study showed that the CLIP staging system is the most stable and optimal model (24). However, none of the above staging systems include MVI status in their staging criteria.

This study aimed to investigate which staging system was the relative optimal one for HCC patients with MVI and to evaluate whether MVI was an independent risk factor in various subgroups of the eight existing staging systems, and attempt to find the basis for integrating MVI into the above staging systems.

## Methods

### Patients

A retrospective study was conducted on consecutive HCC patients who underwent LR with curative intent at the First Affiliated Hospital of Wenzhou Medical University from March 2014 to March 2016 and the Eastern Hepatobiliary Surgery Hospital from February 2014 to January 2015. This study was approved by the Institutional Ethics Committees of the First Affiliated Hospital of Wenzhou Medical University and the Eastern Hepatobiliary Surgery Hospital. As patients’ identities were anonymized, the requirement for informed consent was waived by the Ethics Committees.

The inclusion criteria were patients with (I) HCC confirmed by postoperative histopathology and cytology, (II) well preserved liver function with Child-Pugh class A or B7, (III) LR with R0 status (no gross residual tumor under visual observation, and negative resection margins under microscopy), and (IV) preoperative imaging data of contrast-enhanced magnetic resonance imaging (MRI) of abdomen. The exclusion criteria were patients with (I) extrahepatic metastasis, (II) preoperative radiofrequency ablation, (III) recurrent HCC, (IV) a previous history of other malignancies, and (V) incomplete clinical data.

### Staging Systems

Eight staging systems include BCLC, HKLC, CLIP score, TIS score, LCSGJ, Tokyo score, AJCC TNM 7^th^ edition, and Okuda staging systems (16–21, 25). **Table 1** summarizes the key characteristics of each staging system. The detailed staging criteria are presented in the supplementary material.

**Table 1:** Indicators of the eight staging systems.

	ECOG PS	Child-Pugh	Tumor size	Tumor number	Bilirubin	Portal hypertension	Macrovascular invasion	Extrahepatic metastases	APF	Albumin	Ascites
BCLC	√	√	√	√	√	√	√	√			√
HKLC	√	√	√	√			√	√			
CLIP score		√	Volume	√			√		√		
TIS score		√	Volume						√		
LCSGJ			√	√			√	√			
Tokyo score			√	√	√					√	
AJCC TNM 7^th^			√	√			√	√			
Okuda			Volume		√					√	√

BCLC, Barcelona Clinic Liver Cancer; HKLC, Hong Kong Liver Cancer; CLIP, Cancer of the Liver Italian Program; TIS, Taipei Integrated Scoring System; LCSGJ, Liver Cancer Study Group of Japan; ACJJ, American Joint Cancer Committee; TNM, Tumor-Node-Metastasis.

### Definitions

MVI was defined as “a cancer cell nest with >50 cells in the endothelial vascular lumen under microscopy” (7). In this study, the method to detect MVI was the 7-point sampling protocol (7). Macrovascular invasion, including portal vein tumor thrombus (PVTT) and hepatic vein tumor thrombus (HVTT), was defined as radiological evidence of tumor invasion into the major vasculatures or their main branches. Bile duct tumor thrombus (BDTT) was defined as radiological evidence of tumor invasion into the bile duct.

### Investigations and Hepatectomy

Routine preoperative investigations included blood tests, coagulation profile, liver and kidney functions, hepatitis serology, serum alpha-fetoprotein (AFP), abdominal ultrasound, magnetic resonance imaging (MRI), and computed tomography (CT) scanning. Preoperative diagnosis of HCC was based on the criteria proposed by the AASLD (2). Hepatectomy was performed as previously described (26–28). In both surgical centers in this study, anatomical resection is the first choice for a single tumor, or multiple tumors located in a single liver segment or adjacent segments. For multiple tumors involving the right and left hemilivers, anatomical resection is used for the main tumor, while non-anatomical resection with an adequate resection margin for satellite nodules (29). For patients with an insufficient residual liver volume, non-anatomical resection is used to achieve a negative resection margin. A negative margin was defined as the lack of tumor cells on microscopic examination of the resected margins of the specimen. For patients with combined macrovascular invasion or BDTT, the tumor thrombus would be removed intraoperatively either by thrombectomy or by concomitant extrahepatic bile duct resection (30–32).

### Follow-Up

All patients were regularly followed up in the outpatient clinic once every 1–3 months after discharge from hospital. At each follow-up visit, there were routine medical history taking, physical examination, laboratory blood tests, and abdominal ultrasonography or contrast enhanced CT/MRI. The primary end point of this study was overall survival (OS), which was defined as the time from initial hepatectomy to the date of death or the date of last follow-up. Disease-free survival (DFS) was defined as the time from hepatic resection to the diagnosis of tumor recurrence.

### Statistics

All clinical data were analyzed using SPSS version 25.0 (SPSS Inc., Chicago, IL, USA) or R 4.0 software (http://www.r-project.org/). Survival curves were generated using the Kaplan-Meier method and compared using the log-rank test. Univariate Cox regression analysis was used to evaluate the potential significance of each variable in the entire cohort. All variables that were significantly related to OS (p<0.05) were incorporated into the multivariate Cox regression analysis (backward stepwise selection process, p<0.05). Corrected Akaike information criterion (AICc) was obtained to reveal how staging systems were correlated with the patients’ survival. Homogeneity was measured by Wald χ^2^ test to assess the differences in survival of patients in the same stage within each system (33).

## Results

### Patient Characteristics and Overall Survival

Of 1,198 patients at the First Affiliated Hospital of Wenzhou Medical University and the Eastern Hepatobiliary Surgery Hospital with complete clinicopathological and follow-up data, there were 510 (42.6%) patients with MVI and 688 (58.4%) patients without MVI. **Table 2** summarizes the clinicopathological features of these patients. The median age was 51 years, with the majority of male (83%). Nine hundred ninety-four (83%) patients were HBV positive, 18 (2%) patients had BDTT, and 110 (9%) patients had macrovascular invasion. There were 114 (10%) patients who received neoadjuvant transcatheter arterial chemoembolization (TACE) and 507 (42%) patients who underwent adjuvant TACE. There were some differences at baseline between patients with MVI and those without MVI (**Supplemental Table 1**). The median follow-up for the entire cohort was 34 months.

**Table 2:** Demographic and clinical information of the entire hepatocellular carcinoma cohort.

Variables	All patients (n=1,198)
Age (years, median [interquartile range])	51 (18–83)
Sex (male/female), n (%)	994/204 (83/17)
HBsAg (positive/negative), n (%)	994/204 (83/17)
Antiviral therapy (yes/no), n (%)	118/1,080 (10/90)
Alcoholism (yes/no), n (%)	320/878 (27/73)
Current smoking (yes/no), n (%)	418/780 (35/65)
Diabetes mellitus (yes/no), n (%)	90/1,108 (8/92)
Ascites (present/absent), n (%)	114/1,084 (10/90)
Albumin (g/dl), mean ± SD	41.3 ± 4.8
Bilirubin (mg/dl), mean ± SD	1.0 ± 2.2
Alanine aminotransferase (U/L), mean ± SD	51.8 ± 50.9
Prealbumin (mg/L), mean ± SD	229.4 ± 69.3
Creatinine (mg/dl), mean ± SD	0.6 ± 0.2
Platelet (10^9/L), mean ± SD	158.4 ± 71.4
Alpha-fetoprotein (ng/ml, ≤20/20–400/>400), n (%)	502/312/384 (42/26/32)
Hilar occlusion time (>30/≤30 min), n (%)	59/1,139 (5/95)
Surgical margin (wide/narrow), n (%)	798/400 (67/33)
Varicose veins of gastric fundus (yes/no), n (%)	158/1,040 (13/87)
BDTT (yes/no), n (%)	18/1,180 (2/98)
Macrovascular invasion (yes/no), n (%)	110/1,088 (9/91)
MVI (yes/no), n (%)	510/688 (43/57)
Liver cirrhosis (yes/no), n (%)	804/394 (67/33)
Tumor nodules (1/2/≥3), n (%)	1,066/114/18 (89/10/1)
Maximal tumor diameter (≤2/2–5/>5 cm), n (%)	118/540/540 (10/45/45)
Neoadjuvant TACE (yes/no), n (%)	114/1,084 (10/90)
Adjuvant TACE (yes/no), n (%)	507/691 (42/58)

HR, hazard ratio; CI, confidence interval; HBsAg, hepatitis B surface antigen; min, minutes; BDTT, bile duct tumor thrombus; MVI microvascular invasion; TACE, transcatheter arterial chemoembolization.

Macrovascular invasion including portal vein tumor thrombus (PVTT) and hepatic vein tumor thrombus (HVTT).

### Baseline Predictors of Survival

Univariate regression analysis revealed that sex, antiviral therapy, current smoking, ascites, albumin, alanine aminotransferase (ALT), prealbumin, AFP, varicose veins of gastric fundus, BDTT, macrovascular invasion, MVI, tumor number, and maximal tumor diameter were potential risk factors of survival in HCC patients (**Table 3**). Multivariate regression analysis of these factors showed that current smoking, albumin, prealbumin, AFP, varicose veins of gastric fundus, BDTT, macrovascular invasion, MVI, tumor number, and maximal tumor diameter as independent risk factors of survival of patients with HCC.

**Table 3:** Univariate and multivariate survival analysis in the entire hepatocellular carcinoma cohort.

Overall survival	Number	Univariate analysis			Multivariate analysis		
		HR	95% CI	P value	HR	95% CI	P value
All patients (n=1,198)							
Age (≥65/<65 years)	136/1,062	1.151	0.902–1.468	0.259			
Sex (male/female)	994/204	1.322	1.049–1.665	**0.018**			
HBsAg (positive/negative)	994/204	0.904	0.736–1.111	0.337			
Antiviral therapy (yes/no)	118/1,080	0.685	0.507–0.926	**0.014**			
Alcoholism (yes/no)	320/878	1.053	0.881–1.258	0.573			
Current smoking (yes/no)	418/780	1.323	1.123–1.560	**0.001**	1.310	1.108–1.549	0.002
Diabetes mellitus (yes/no)	90/1,108	0.964	0.717–1.294	0.805			
Ascites (present/absent)	114/1,084	1.363	1.059–1.755	**0.016**			
Albumin (<3.5/≥3.5 g/dl)	56/1,142	1.717	1.236–2.385	**0.001**	1.718	1.231–2.396	0.001
Bilirubin (≥1/<1 mg/dl)	328/870	1.125	0.941–1.344	0.196			
Alanine aminotransferase (>40/≤40 U/L)	552/646	1.359	1.157–1.596	**<0.001**			
Prealbumin (<280/≥280 mg/L)	988/210	1.401	1.117–1.757	**0.004**	1.292	1.029–1.623	0.028
Creatinine (≥1/<1 mg/dl)	20/1,178	1.055	0.564–1.971	0.868			
Platelet (<100/≥100 *10^9/L)	260/938	1.034	0.854–1.252	0.732			
Alpha-fetoprotein (≥20/<20 ng/ml)	696/502	1.444	1.221–1.707	**<0.001**	1.314	1.103–1.566	0.002
Hilar occlusion time (>30/≤30 min)	59/1,139	1.330	0.949–1.863	0.098			
Surgical margin (wide/narrow)	798/400	1.137	0.955–1.353	0.148			
Varicose veins of gastric fundus (yes/no)	158/1,040	1.444	1.156–1.804	**0.001**	1.466	1.166–1.843	0.001
BDTT (yes/no)	18/1180	1.875	1.104–3.187	**0.020**	2.761	1.615–4.720	<0.001
Macrovascular invasion (yes/no)	110/1,088	4.129	3.292–5.177	**<0.001**	2.595	2.012–3.347	<0.001
MVI (yes/no)	510/688	2.089	1.777–2.456	**<0.001**	1.518	1.267–1.819	<0.001
Liver cirrhosis (yes/no)	804/394	1.005	0.847–1.192	0.957			
Tumor nodules (multiple/single)	132/1,066	1.739	1.389–2.177	**<0.001**	1.628	1.289–2.056	<0.001
Maximal tumor diameter (>5/≤5 cm)	540/658	2.455	2.083–2.894	**<0.001**	2.097	1.768–2.488	<0.001
Neoadjuvant TACE (yes/no)	114/1,084	1.137	0.867–1.491	0.354			
Adjuvant TACE (yes/no)	507/691	0.865	0.735–1.019	0.083			

HR, hazard ratio; CI, confidence interval; HBsAg, hepatitis B surface antigen; min, minutes; BDTT, bile duct tumor thrombus; MVI microvascular invasion; TACE, transcatheter arterial chemoembolization.

Macrovascular invasion including portal vein tumor thrombus (PVTT) and hepatic vein tumor thrombus (HVTT).

Bold values provided mean P < 0.05.

### Prognostic Performance of the Eight Staging Systems

**Figure 1:**
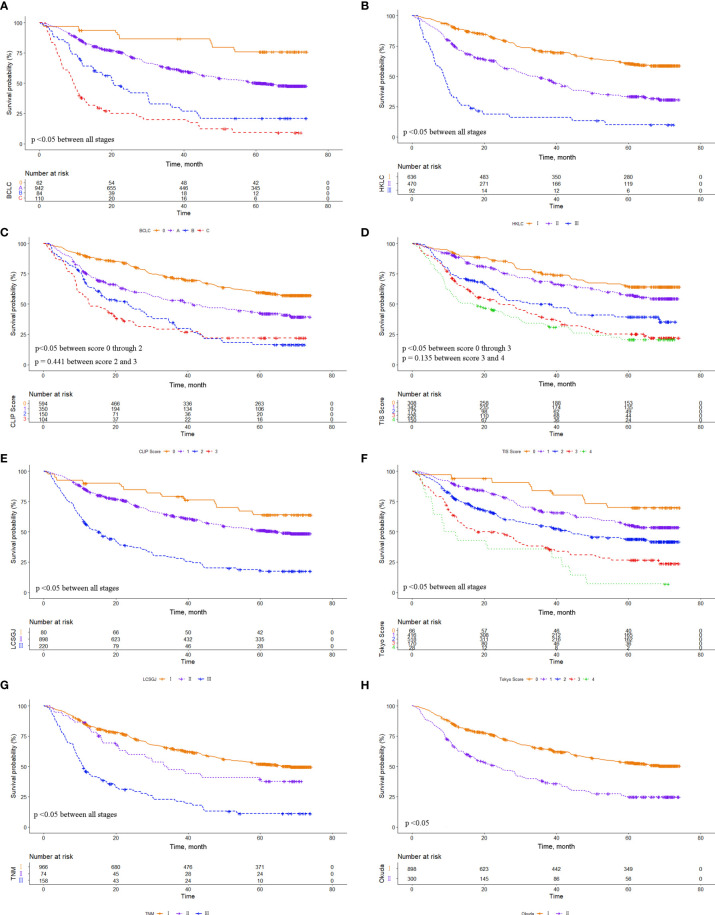
Comparison of overall survival distributions by **(A)** Barcelona Clinic Liver Cancer, **(B)** Hong Kong Liver Cancer, **(C)** Cancer of the Liver Italian Program, **(D)** Taipei Integrated Scoring, **(E)** Tumor-Node-Metastasis by Liver Cancer Study Group of Japan, **(F)** Tokyo, **(G)** Tumor-Node-Metastasis by American Joint Cancer Committee 7^th^ edition, and **(H)** Okuda staging systems in the entire cohort.

**Figure 2:**
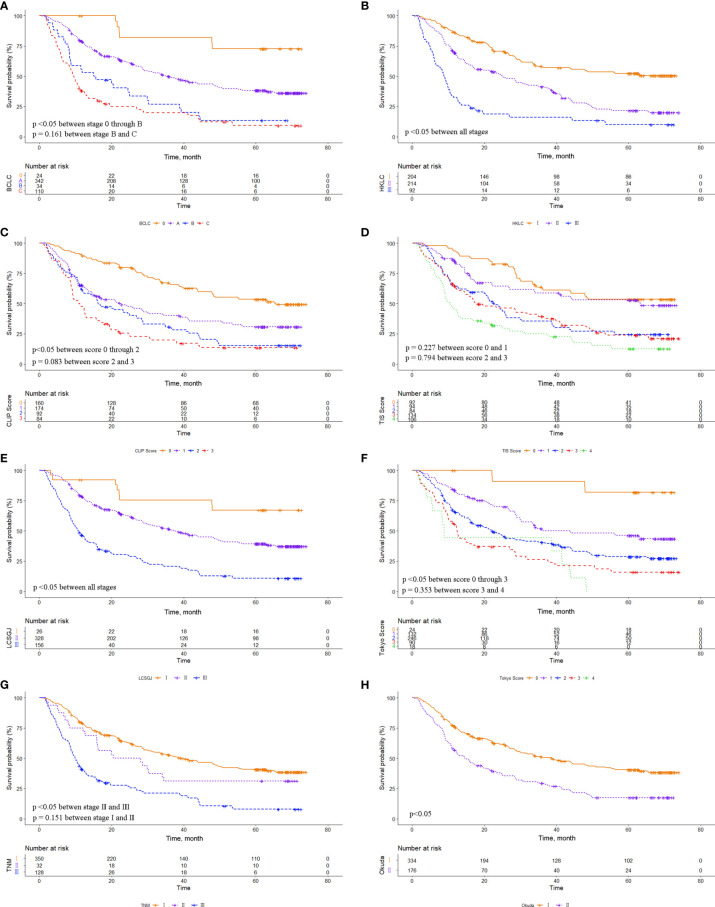
Comparison of overall survival distributions by **(A)** Barcelona Clinic Liver Cancer, **(B)** Hong Kong Liver Cancer, **(C)** Cancer of the Liver Italian Program, **(D)** Taipei Integrated Scoring, **(E)** Tumor-Node-Metastasis by Liver Cancer Study Group of Japan, **(F)** Tokyo, **(G)** Tumor-Node-Metastasis by American Joint Cancer Committee 7^th^ edition, and **(H)** Okuda staging systems in the microvascular invasion (MVI) cohort.

The eight common HCC staging systems were evaluated respectively with Kaplan-Meier survival analysis. In the entire cohort, significant differences in survival distribution were observed for all stages of BCLC, HKLC, CLIP score, TIS score, LCSGJ, Tokyo score, TNM, and Okuda staging system (p<0.05). There were no significant survival differences between CLIP scores 2 and 3 (p = 0.441), and between TIS scores 3 and 4 (p = 0.135) (**Figure 1**). The role of CLIP score, TIS score, and Tokyo score in discriminating DFS was limited (**Supplementary Figure 1**). In the MVI cohort, significant differences in survival distribution were also found for all stages of BCLC, HKLC, CLIP score, TIS score, LCSGJ, Tokyo score, TNM, and Okuda staging system (p<0.05). There were no significant survival differences between BCLC stages B and C (p=0.161), CLIP scores 2 and 3 (p = 0.083), TIS scores 0 and 1 (p = 0.227), TIS scores 2 and 3 (p = 0.794), Tokyo scores 3 and 4 (p=0.353), and TNM stages I and II (p=0.151) (**Figure 2**). The discrimination ability for DFS of the eight staging systems in patients with MVI is detailed in **Supplementary Figure 2**.

The prognostic performance of the eight staging systems is shown in **Table 4**. In all patient cohorts, the HKLC system provided the lowest AICc value and the highest homogeneity, followed by the TIS and BCLC system. In the MVI cohorts, the HKLC was consistently associated with the lowest AICc value and the highest homogeneity.

**Table 4:** Comparison of prognostic performance among eight staging systems.

Model	Homogeneity (Wald χ2)	Corrected Akaike information criteria (AICc)
All patients (n=1,198)		
BCLC	196.4	7,742.260
HKLC	221.2	7,717.447
CLIP	154.1	7,766.160
TIS	168.9	7,742.071
LCSGJ	143.2	7,785.127
Tokyo	108.0	7,809.815
AJCC TNM 7^th^	172.1	7,775.350
Okuda	87.07	7,830.135
MVI patients (n=510)		
BCLC	86.6	3,565.216
HKLC	102.0	3,562.844
CLIP	74.7	3,583.413
TIS	72.2	3,586.370
LCSGJ	81.1	3,575.127
Tokyo	50.9	3,591.941
AJCC TNM 7^th^	82.0	3,589.966
Okuda	36.3	3,625.387

BCLC, Barcelona Clinic Liver Cancer; HKLC, Hong Kong Liver Cancer; CLIP, Cancer of the Liver Italian Program; TIS, Taipei Integrated Scoring System; LCSGJ, Liver Cancer Study Group of Japan; AJCC, American Joint Cancer Committee; TNM, Tumor-Node-Metastasis; MVI, microvascular invasion.

### The Prognostic Value of MVI in Subgroups

In BCLC staging system, there was no significant difference in survival between patients with and without MVI in stages 0 (p=0.75) and B (p=0.1), and patients with MVI had worse survival in stage A (p<0.001). Notably, we detected MVI in all patients with macrovascular invasion. In HKLC staging system, patients with MVI had worse survival in stages I and II (p<0.001). In CLIP scoring system, there was no significant difference in score 2 (p=0.17), and patients with MVI had worse survival in scores 0, 1, and 3 (p<0.05) (**Supplementary Figure 3**). In TIS scoring system, there was no significant difference in scores 1 (p=0.062) and 3 (p=0.28), and patients with MVI had worse survival in scores 0, 2, and 4 (p<0.05). In LCSGJ staging system, there was no significant difference in stage I (p=0.78), and patients with MVI had worse survival in stages II and III (p<0.001) (**Supplementary Figure 4**). In Tokyo scoring system, there was no significant difference in scores 0 (p=0.12) and 4 (p=0.56), and patients with MVI had worse survival in scores 1, 2, and 3 (p<0.001). In TNM staging system, there was no significant difference in stage II (p=0.087), and patients with MVI had worse survival in stages I and III (p<0.05) (**Supplementary Figure 5**). And in Okuda staging system, patients with MVI had worse survival for stages I and II (p<0.001) (**Supplementary Figure 6**).

We adjusted the effect of MVI on overall survival in all subgroups. The adjusted HR was calculated by multivariable COX regression model with covariates listed in **Supplementary Table 1**. There was no significant difference in CLIP score 0 (adjusted HR 1.273, 95% CI 0.942–1.720, p=0.116), while patients with MVI had worse survival in CLIP scores 2 (adjusted HR 1.868, 95% CI 1.111–3.141, p=0.018) and TIS score 1 (adjusted HR 1.675, 95% CI 1.105–2.539, p=0.015).

## Discussion

MVI is one of the most important prognostic factors in patients with HCC (8–12). At present, there are many models to predict the occurrence of MVI before operation and to assess the prognosis of MVI patients (26, 34, 35). The predictors in these models include tumor diameter, tumor number, and other factors that have also existed in the current HCC staging systems. Therefore, whether the current HCC staging systems are able to distinguish the prognosis of MVI patients and whether the prognostic significance of MVI in different subgroups of HCC staging systems are worth exploring. In this study, we collected data of HCC patients who underwent radical surgery from two high-volume clinical centers. Possible prognostic factors were examined, and eight staging systems were evaluated. We confirmed that the key prognostic factors of HCC include current smoking, albumin, prealbumin, AFP, varicose veins of gastric fundus, BDTT, macrovascular invasion, MVI, tumor number, and maximal tumor diameter. We also demonstrated that among the eight staging systems currently used, HKLC is the best prognostic model and provides a better prognostic prediction ability. The results were consistent in the MVI cohort.

In this study, important prognostic factors for HCC were identified. There is no doubt that patients’ bad living habits, such as smoking, will reduce their survival (36, 37). Albumin and prealbumin levels are closely related to the severity of liver cirrhosis, and it is not surprising that they can predict adverse outcomes in patients with HCC (24). Alpha-fetoprotein level, macrovascular invasion, MVI, and tumor load have been considered as important prognostic indexes (38, 39). Consistent with our previous studies, our results showed that gastric varices and BDTT reflect poor survival outcomes in HCC patients (26, 40).

Using Kaplan-Meier survival analysis, we showed that all eight HCC staging systems were associated with a trend of gradually decreasing survival from early to advanced stages. The survival difference was obvious for each system in the whole cohort. However, the survival difference was insignificant between CLIP score 2/3, TIS score 3/4, which may be related to the fact that our included patients all underwent LR, and most patients were subject to HBV infection. In the MVI cohort, there were no significant survival differences among BCLC stage B/C, CLIP score 2/3, TIS score 0/1, TIS score 2/3, Tokyo score 2/3, and TNM 7^th^ stage I/II, which may be attributed to the fact that MVI decreased overall patient survival, and the prognostic value of MVI differed among the various subgroups.

In subgroup analysis, we found that patients in BLCL stages 0 and B, CLIP score 0, TIS scores 3, LCSGJ stage I, Tokyo scores 0 and 4, and TNM 7^th^ stage II, with or without MVI, had no significant impact on patients’ survival. To the best of our knowledge, this phenomenon of different prognostic significance of MVI among various subgroups has been previously documented. Huang et al. reported that the clinical value of microvascular invasion in HCC patients at BCLC stage 0 or B was limited (12). Different surgical approaches and various resection ranges of patients may markedly affect the MVI status in the residual liver, and there are some other clinical factors that will affect the prognosis of HCC patients at different stages. Chan et al. refined the seventh edition of AJCC TNM staging and incorporated MVI status into the T staging, and the updated T staging can better stratify HCC patients into subsets with distinct long-term prognosis (41). In addition, the TNM staging of the eighth edition AJCC describes vascular invasion as “For pathological classification, vascular invasion includes gross as well as microscopic involvement of vessels.” These data indicate that MVI can influence the accuracy of the existing HCC staging systems, and imply that the modification of the existing HCC staging system is imperative.

Several limitations of this study must be acknowledged. First, this is a retrospective study with its inherent defects. Second, because MVI can only be diagnosed postoperatively, it is not allowed to discuss this for patients who are not candidates for surgery. Third, the used method to diagnose MVI was the 7-point sampling protocol, which can lead to under-diagnosis of MVI. Fourth, this study was conducted in China, where HBV infection rate is high, and HBV is associated with a high incidence of MVI (42–45). The results of this study may not be applicable to HCC patients with other etiologic factors.

In summary, our results suggested that the HKLC staging system is the most accurate prognostic model among the eight commonly used HCC staging systems. In each subgroup of the staging systems, although MVI showed different prognostic value, it generally exhibited poor survival outcomes. At the same time, our results showed that MVI may be needed to be incorporated into the current HCC staging systems as one of the grading criteria.

## Data Availability Statement

The raw data supporting the conclusions of this article will be made available by the authors, without undue reservation.

## Ethics Statement

The studies involving human participants were reviewed and approved by the Institutional Ethics Committees of the First Affiliated Hospital of Wenzhou Medical University and the Eastern Hepatobiliary Surgery Hospital. Written informed consent for participation was not required for this study in accordance with the national legislation and the institutional requirements.

## Authors Contributions

Conception and Design: S-QC, Y-FS, Y-JX, and KW. Financial Support: S-QC Y-FS, and KW. Provision of Study Materials or Patients: Y-JX, KW, Y-TZ, and H-MY. Collection and Assembly of Data: Y-JX, KW, Y-QC, and W-JW. Data Analysis and Interpretation: Y-JX, KW, and Y-TZ. Manuscript Writing: All authors. Final Approval of Manuscript: All authors. All authors contributed to the article and approved the submitted version.

## Funding

This work was supported by the Clinical Research Plan of SHDC (No. SHDC2020CR1004A), the State Key Program of National Natural Science Foundation of China (No: 81730097), the National Natural Science Foundation of China (No: 82072618 and 81770630), and the Science and Technology Commission Foundation of Shanghai Municipality (No: 19411967300).

## Conflict of Interest

The authors declare that the research was conducted in the absence of any commercial or financial relationships that could be construed as a potential conflict of interest.

## Publisher’s Note

All claims expressed in this article are solely those of the authors and do not necessarily represent those of their affiliated organizations, or those of the publisher, the editors and the reviewers. Any product that may be evaluated in this article, or claim that may be made by its manufacturer, is not guaranteed or endorsed by the publisher.

## Supplementary Material

Supplementary Figure 1 Comparison of disease-free survival distributions by **(A)** Barcelona Clinic Liver Cancer, **(B)** Hong Kong Liver Cancer, **(C)** Cancer of the Liver Italian Program, **(D)** Taipei Integrated Scoring, **(E)** Tumor-Node-Metastasis by Liver Cancer Study Group of Japan, **(F)** Tokyo, **(G)** Tumor-Node-Metastasis by American Joint Cancer Committee 7^th^ edition, and **(H)** Okuda staging systems in the entire cohort.

Supplementary Figure 2 Comparison of disease-free survival distributions by **(A)** Barcelona Clinic Liver Cancer, **(B)** Hong Kong Liver Cancer, **(C)** Cancer of the Liver Italian Program, **(D)** Taipei Integrated Scoring, **(E)** Tumor-Node-Metastasis by Liver Cancer Study Group of Japan, **(F)** Tokyo, **(G)** Tumor-Node-Metastasis by American Joint Cancer Committee 7^th^ edition, and **(H)** Okuda staging systems in the microvascular invasion (MVI) cohort.

Supplementary Figure 3 Cumulative overall survival (OS) curves of patients with or without microvascular invasion (MVI). **(A)** BCLC stage 0, **(B)** BCLC stage A, **(C)** BCLC stage B, **(D)** HKLC stage I, **(E)** HKLC stage II, **(F)** CLIP score 0, **(G)** CLIP score 1, **(H)** CLIP score 2, **(I)** CLIP score 3. BCLC, Barcelona Clinic Liver Cancer; HKLC, Hong Kong Liver Cancer; CLIP, Cancer of the Liver Italian Program.

Supplementary Figure 4 Cumulative overall survival (OS) curves of patients with or without microvascular invasion (MVI). **(A)** TIS score 0, **(B)** TIS score 1, **(C)** TIS score 2, **(D)** TIS score 3, **(E)** TIS score 4, **(F)** LCSGJ stage I, **(G)** LCSGJ stage II, **(H)** LCSGJ stage III. TIS, Taipei Integrated Scoring System; LCSGJ, Liver Cancer Study Group of Japan.

Supplementary Figure 5 Cumulative overall survival (OS) curves of patients with or without microvascular invasion (MVI). **(A)** Tokyo score 0, **(B)** Tokyo score 1, **(C)** Tokyo score 2, **(D)** Tokyo score 3, **(E)** Tokyo score 4, **(F)** AJCC TNM 7^th^ stage I, **(G)** AJCC TNM 7^th^ stage II, **(H)** AJCC TNM 7^th^ stage III. AJCC, American Joint Cancer Committee; TNM, Tumor-Node-Metastasis.

Supplementary Figure 6 Cumulative overall survival (OS) curves of patients with or without microvascular invasion (MVI). **(A)** Okuda stage I, **(B)** Okuda stage II.

## Abbreviations

HCC, hepatocellular carcinoma; LR, liver resection; MVI, microvascular invasion; BCLC, Barcelona Clinic Liver Cancer; EASL, European Association for the Study of the Liver; AASLD, American Association for the Study of Liver Diseases; HKLC, Hong Kong Liver Cancer; CLIP, Cancer of the Liver Italian Program; TIS, Taipei Integrated Scoring System; TNM, Tumor-Node-Metastasis; LCSGJ, Liver Cancer Study Group of Japan; AJCC, American Joint Cancer Committee; PVTT, portal vein tumor thrombus; HVTT, hepatic vein tumor thrombus; BDTT, bile duct tumor thrombus; AFP, alpha-fetoprotein; MRI, magnetic resonance imaging; CT, computed tomography; OS, overall survival; AICc, Corrected Akaike information criterion; ALT, alanine aminotransferase.

## References

[1] Siegel RL Miller KD Jemal A. Cancer Statistics, 2020. CA Cancer J Clin (2020) 70(1):7–30. doi: 10.3322/caac.21590 31912902

[2] Marrero JA Kulik LM Sirlin CB Zhu AX Finn RS Abecassis MM. Diagnosis, Staging, and Management of Hepatocellular Carcinoma: 2018 Practice Guidance by the American Association for the Study of Liver Diseases. Hepatology (2018) 68(2):723–50. doi: 10.1002/hep.29913 29624699

[3] European Association for the Study of the Liver Electronic address eee, European Association for the Study of the L. EASL Clinical Practice Guidelines: Management of Hepatocellular Carcinoma. J Hepatol (2018) 69(1):182–236. doi: 10.1016/j.jhep.2018.03.019 29628281

[4] Xie DY Ren ZG Zhou J Fan J Gao Q. 2019 Chinese Clinical Guidelines for the Management of Hepatocellular Carcinoma: Updates and Insights. Hepatobiliary Surg Nutr (2020) 9(4):452–63. doi: 10.21037/hbsn-20-480 PMC7423548 32832496

[5] Grazi GL Ercolani G Pierangeli F Del Gaudio M Cescon M Cavallari A. Improved Results of Liver Resection for Hepatocellular Carcinoma on Cirrhosis Give the Procedure Added Value. Ann Surg (2001) 234(1):71–8. doi: 10.1097/00000658-200107000-00011 PMC1421950 11420485

[6] Lim KC Chow PK Allen JC Siddiqui FJ Chan ES Tan SB. Systematic Review of Outcomes of Liver Resection for Early Hepatocellular Carcinoma Within the Milan Criteria. Br J Surg (2012) 99(12):1622–9. doi: 10.1002/bjs.8915 23023956

[7] Cong WM Bu H Chen J Dong H Zhu YY Feng LH. Practice Guidelines for the Pathological Diagnosis of Primary Liver Cancer: 2015 Update. World J Gastroenterol (2016) 22(42):9279–87. doi: 10.3748/wjg.v22.i42.9279 PMC5107692 27895416

[8] Rodriguez-Peralvarez M Luong TV Andreana L Meyer T Dhillon AP Burroughs AK. A Systematic Review of Microvascular Invasion in Hepatocellular Carcinoma: Diagnostic and Prognostic Variability. Ann Surg Oncol (2013) 20(1):325–39. doi: 10.1245/s10434-012-2513-1 23149850

[9] Lim KC Chow PK Allen JC Chia GS Lim M Cheow PC. Microvascular Invasion is a Better Predictor of Tumor Recurrence and Overall Survival Following Surgical Resection for Hepatocellular Carcinoma Compared to the Milan Criteria. Ann Surg (2011) 254(1):108–13. doi: 10.1097/SLA.0b013e31821ad884 21527845

[10] Mazzaferro V Llovet JM Miceli R Bhoori S Schiavo M Mariani L. Predicting Survival After Liver Transplantation in Patients With Hepatocellular Carcinoma Beyond the Milan Criteria: A Retrospective, Exploratory Analysis. Lancet Oncol (2009) 10(1):35–43. doi: 10.1016/S1470-2045(08)70284-5 19058754

[11] Sumie S Kuromatsu R Okuda K Ando E Takata A Fukushima N. Microvascular Invasion in Patients With Hepatocellular Carcinoma and its Predictable Clinicopathological Factors. Ann Surg Oncol (2008) 15(5):1375–82. doi: 10.1245/s10434-008-9846-9 18324443

[12] Huang C Zhu XD Ji Y Ding GY Shi GM Shen YH. Microvascular Invasion has Limited Clinical Values in Hepatocellular Carcinoma Patients at Barcelona Clinic Liver Cancer (BCLC) Stages 0 or B. BMC Cancer (2017) 17(1):58. doi: 10.1186/s12885-017-3050-x 28095820 PMC5240309

[13] Shindoh J Andreou A Aloia TA Zimmitti G Lauwers GY Laurent A. Microvascular Invasion Does Not Predict Long-Term Survival in Hepatocellular Carcinoma Up to 2 Cm: Reappraisal of the Staging System for Solitary Tumors. Ann Surg Oncol (2013) 20(4):1223–9. doi: 10.1245/s10434-012-2739-y PMC3856190 23179993

[14] Shen J Wen J Li C Wen T Yan L Li B. The Prognostic Value of Microvascular Invasion in Early-Intermediate Stage Hepatocelluar Carcinoma: A Propensity Score Matching Analysis. BMC Cancer (2018) 18(1):278. doi: 10.1186/s12885-018-4196-x 29530006 PMC5848587

[15] Dhir M Melin AA Douaiher J Lin C Zhen WK Hussain SM. A Review and Update of Treatment Options and Controversies in the Management of Hepatocellular Carcinoma. Ann Surg (2016) 263(6):1112–25. doi: 10.1097/SLA.0000000000001556 26813914

[16] Yau T Tang VY Yao TJ Fan ST Lo CM Poon RT. Development of Hong Kong Liver Cancer Staging System With Treatment Stratification for Patients With Hepatocellular Carcinoma. Gastroenterology (2014) 146(7):1691–700 e3. doi: 10.1053/j.gastro.2014.02.032 24583061

[17] Tateishi R Yoshida H Shiina S Imamura H Hasegawa K Teratani T. Proposal of a New Prognostic Model for Hepatocellular Carcinoma: An Analysis of 403 Patients. Gut (2005) 54(3):419–25. doi: 10.1136/gut.2003.035055 PMC1774402 15710994

[18] Kudo M Chung H Osaki Y. Prognostic Staging System for Hepatocellular Carcinoma (CLIP Score): Its Value and Limitations, and a Proposal for a New Staging System, the Japan Integrated Staging Score (JIS Score). J Gastroenterol (2003) 38(3):207–15. doi: 10.1007/s005350300038 12673442

[19] Chevret S Trinchet J-C Mathieu D Rached AA Beaugrand M Chastang C. A New Prognostic Classification for Predicting Survival in Patients With Hepatocellular Carcinoma. J Hepatol (1999) 31(1):133–41. doi: 10.1016/s0168-8278(99)80173-1 10424293

[20] Hsu CY Huang YH Hsia CY Su CW Lin HC Loong CC. A New Prognostic Model for Hepatocellular Carcinoma Based on Total Tumor Volume: The Taipei Integrated Scoring System. J Hepatol (2010) 53(1):108–17. doi: 10.1016/j.jhep.2010.01.038 20451283

[21] Okuda K Obata H Nakajima Y Ohtsuki T Okazaki N Ohnishi K. Prognosis of Primary Hepatocellular Carcinoma. Hepatology (1984) 4(1 Suppl):3S–6S. doi: 10.1002/hep.1840040703 6319264

[22] Chapiro J Geschwind JF. Hepatocellular Carcinoma: Have We Finally Found the Ultimate Staging System for HCC? Nat Rev Gastroenterol Hepatol (2014) 11(6):334–6. doi: 10.1038/nrgastro.2014.67 24798199

[23] Subramaniam S Kelley RK Venook AP. A Review of Hepatocellular Carcinoma (HCC) Staging Systems. Chin Clin Oncol (2013) 2(4):33. doi: 10.3978/j.issn.2304-3865.2013.07.05 25841912

[24] Liu PH Hsu CY Hsia CY Lee YH Su CW Huang YH. Prognosis of Hepatocellular Carcinoma: Assessment of Eleven Staging Systems. J Hepatol (2016) 64(3):601–8. doi: 10.1016/j.jhep.2015.10.029 26551516

[25] Bruix J Sherman M. Practice Guidelines Committee AAftSoLD. Management of Hepatocellular Carcinoma. Hepatology (2005) 42(5):1208–36. doi: 10.1002/hep.20933 16250051

[26] Zhang XP Wang K Wei XB Li LQ Sun HC Wen TF. An Eastern Hepatobiliary Surgery Hospital Microvascular Invasion Scoring System in Predicting Prognosis of Patients With Hepatocellular Carcinoma and Microvascular Invasion After R0 Liver Resection: A Large-Scale, Multicenter Study. Oncologist (2019) 24(12):e1476–e88. doi: 10.1634/theoncologist.2018-0868 PMC6975940 31138726

[27] Sun JJ Wang K Zhang CZ Guo WX Shi J Cong WM. Postoperative Adjuvant Transcatheter Arterial Chemoembolization After R0 Hepatectomy Improves Outcomes of Patients Who Have Hepatocellular Carcinoma With Microvascular Invasion. Ann Surg Oncol (2016) 23(4):1344–51. doi: 10.1245/s10434-015-5008-z 26714945

[28] Shi M Guo RP Lin XJ Zhang YQ Chen MS Zhang CQ. Partial Hepatectomy With Wide Versus Narrow Resection Margin for Solitary Hepatocellular Carcinoma: A Prospective Randomized Trial. Ann Surg (2007) 245(1):36–43. doi: 10.1097/01.sla.0000231758.07868.71 17197963 PMC1867934

[29] Zhang YF Zhou J Wei W Zou RH Chen MS Lau WY. Intermediate-Stage Hepatocellular Carcinoma Treated With Hepatic Resection: The NSP Score as an Aid to Decision-Making. Br J Cancer (2016) 115(9):1039–47. doi: 10.1038/bjc.2016.301 PMC5117793 27701389

[30] Chen XP Qiu FZ Wu ZD Zhang ZW Huang ZY Chen YF. Effects of Location and Extension of Portal Vein Tumor Thrombus on Long-Term Outcomes of Surgical Treatment for Hepatocellular Carcinoma. Ann Surg Oncol (2006) 13(7):940–6. doi: 10.1245/ASO.2006.08.007 16788755

[31] Kokudo T Hasegawa K Matsuyama Y Takayama T Izumi N Kadoya M. Liver Resection for Hepatocellular Carcinoma Associated With Hepatic Vein Invasion: A Japanese Nationwide Survey. Hepatology (2017) 66(2):510–7. doi: 10.1002/hep.29225 28437844

[32] Kasai Y Hatano E Seo S Taura K Yasuchika K Uemoto S. Hepatocellular Carcinoma With Bile Duct Tumor Thrombus: Surgical Outcomes and the Prognostic Impact of Concomitant Major Vascular Invasion. World J Surg (2015) 39(6):1485–93. doi: 10.1007/s00268-015-2985-9 25651961

[33] Pourhoseingholi MA Hajizadeh E Moghimi Dehkordi B Safaee A Abadi A Zali MR. Comparing Cox Regression and Parametric Models for Survival of Patients With Gastric Carcinoma. Asian Pac J Cancer Prev (2007) 8(3):412–6. 18159979

[34] Li X Huang H Yu X Chen P Ouyang J Huang B. A Novel Prognostic Nomogram Based on Microvascular Invasion and Hematological Biomarkers to Predict Survival Outcome for Hepatocellular Carcinoma Patients. Surg Oncol (2020) 33:51–7. doi: 10.1016/j.suronc.2020.01.006 32561099

[35] Wang ZX Peng W Zhang XY Wen TF Li C. Prognostic Significance of Postoperative Change of PALBI Grade for Patients With Hepatocellular Carcinoma After Hepatectomy. Medicine (Baltimore) (2021) 100(11):e24476. doi: 10.1097/MD.0000000000024476 33725934 PMC7982202

[36] Soerjomataram I Bray F. Planning for Tomorrow: Global Cancer Incidence and the Role of Prevention 2020-2070. Nat Rev Clin Oncol (2021) 18(10):663–72. doi: 10.1038/s41571-021-00514-z 34079102

[37] Rutledge SM Asgharpour A. Smoking and Liver Disease. Gastroenterol Hepatol (N Y) (2020) 16(12):617–25. PMC8132692 34035697

[38] Toyoda H Kumada T Tada T Yama T Mizuno K Sone Y. Differences in the Impact of Prognostic Factors for Hepatocellular Carcinoma Over Time. Cancer Sci (2017) 108(12):2438–44. doi: 10.1111/cas.13406 PMC5715354 28945309

[39] Zhang LX Luo PQ Chen L Song DD Xu AM Xu P. Model to Predict Overall Survival in Patients With Hepatocellular Carcinoma After Curative Hepatectomy. Front Oncol (2020) 10:537526. doi: 10.3389/fonc.2020.537526 33747893 PMC7977285

[40] Sun J Wu J Liu C Shi J Wei Y Zhou J. Typing of Biliary Tumor Thrombus Influences the Prognoses of Patients With Hepatocellular Carcinoma. Cancer Biol Med (2021) 18(3):808–15. doi: 10.20892/j.issn.2095-3941.2020.0202 PMC8330528 34021538

[41] Chan AC Fan ST Poon RT Cheung TT Chok KS Chan SC. Evaluation of the Seventh Edition of the American Joint Committee on Cancer Tumour-Node-Metastasis (TNM) Staging System for Patients Undergoing Curative Resection of Hepatocellular Carcinoma: Implications for the Development of a Refined Staging System. HPB (Oxford) (2013) 15(6):439–48. doi: 10.1111/j.1477-2574.2012.00617.x PMC3664048 23659567

[42] Lei Z Li J Wu D Xia Y Wang Q Si A. Nomogram for Preoperative Estimation of Microvascular Invasion Risk in Hepatitis B Virus-Related Hepatocellular Carcinoma Within the Milan Criteria. JAMA Surg (2016) 151(4):356–63. doi: 10.1001/jamasurg.2015.4257 26579636

[43] Wei X Li N Li S Shi J Guo W Zheng Y. Hepatitis B Virus Infection and Active Replication Promote the Formation of Vascular Invasion in Hepatocellular Carcinoma. BMC Cancer (2017) 17(1):304. doi: 10.1186/s12885-017-3293-6 28464845 PMC5414329

[44] Xu J Liu H Chen L Wang S Zhou L Yun X. Hepatitis B Virus X Protein Confers Resistance of Hepatoma Cells to Anoikis by Up-Regulating and Activating P21-Activated Kinase 1. Gastroenterology (2012) 143(1):199–212 e4. doi: 10.1053/j.gastro.2012.03.053 22484303

[45] Yoo YG Na TY Seo HW Seong JK Park CK Shin YK. Hepatitis B Virus X Protein Induces the Expression of MTA1 and HDAC1, Which Enhances Hypoxia Signaling in Hepatocellular Carcinoma Cells. Oncogene (2008) 27(24):3405–13. doi: 10.1038/sj.onc.1211000 18264140

