# Supplementary figures and images for: Prognostic Value of Microvascular Invasion in Eight Existing Staging Systems for Hepatocellular Carcinoma: A Bi-Centeric Retrospective Cohort Study
Source: Front Oncol. 2021 Dec 16;11:726569. doi: 10.3389/fonc.2021.726569 (PMC8716381; doi:10.3389/fonc.2021.726569)

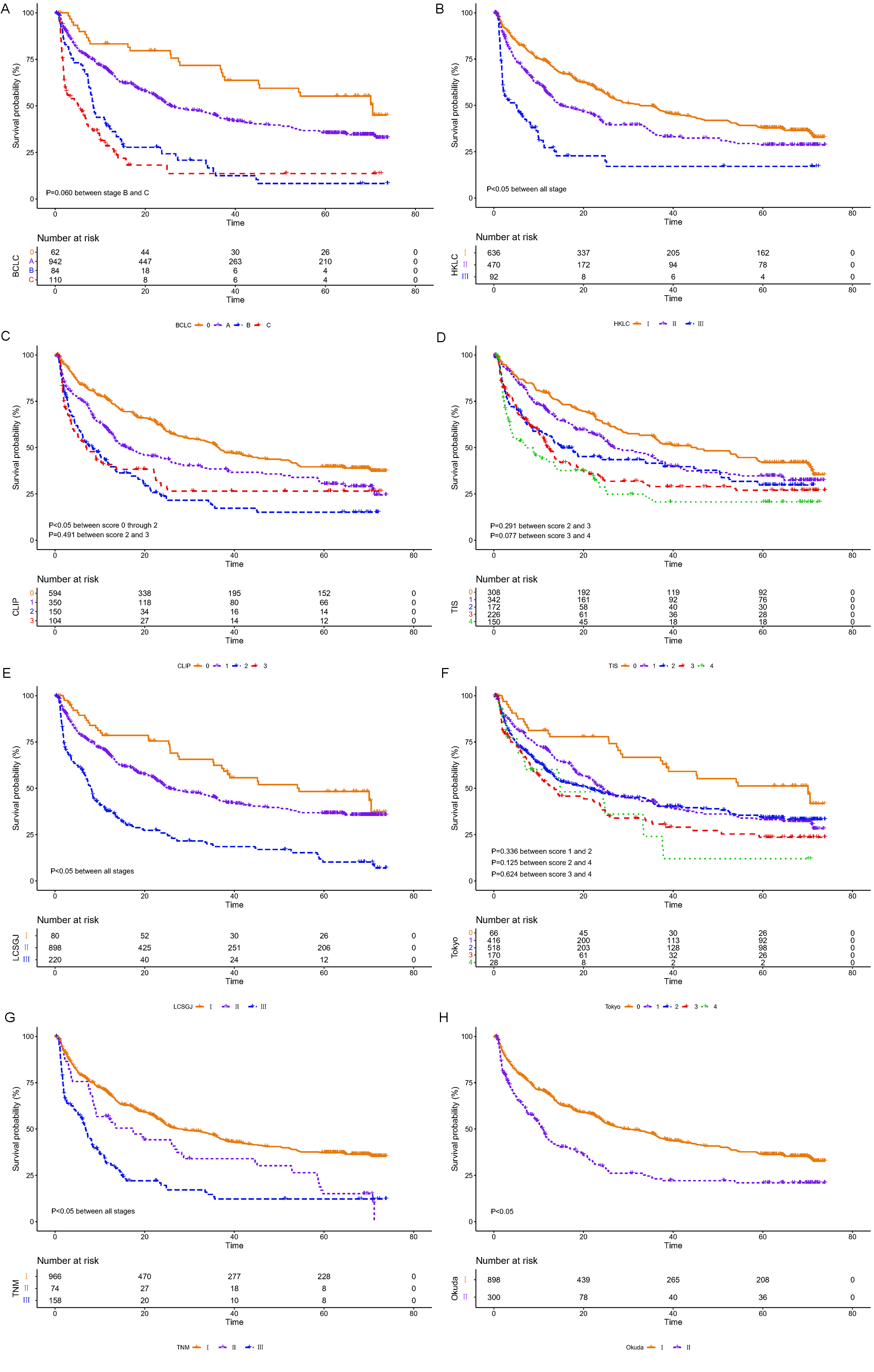

Supplement: Supplementary Figure 1 — Comparison of disease-free survival distributions by (A) Barcelona Clinic Liver Cancer, (B) Hong Kong Liver Cancer, (C) Cancer of the Liver Italian Program, (D) Taipei Integrated Scoring, (E) Tumor-Node-Metastasis by Liver Cancer Study Group of Japan, (F) Tokyo, (G) Tumor-Node-Metastasis by American Joint Cancer Committee 7th edition, and (H) Okuda staging systems in the entire cohort. [file Image_1.tif]

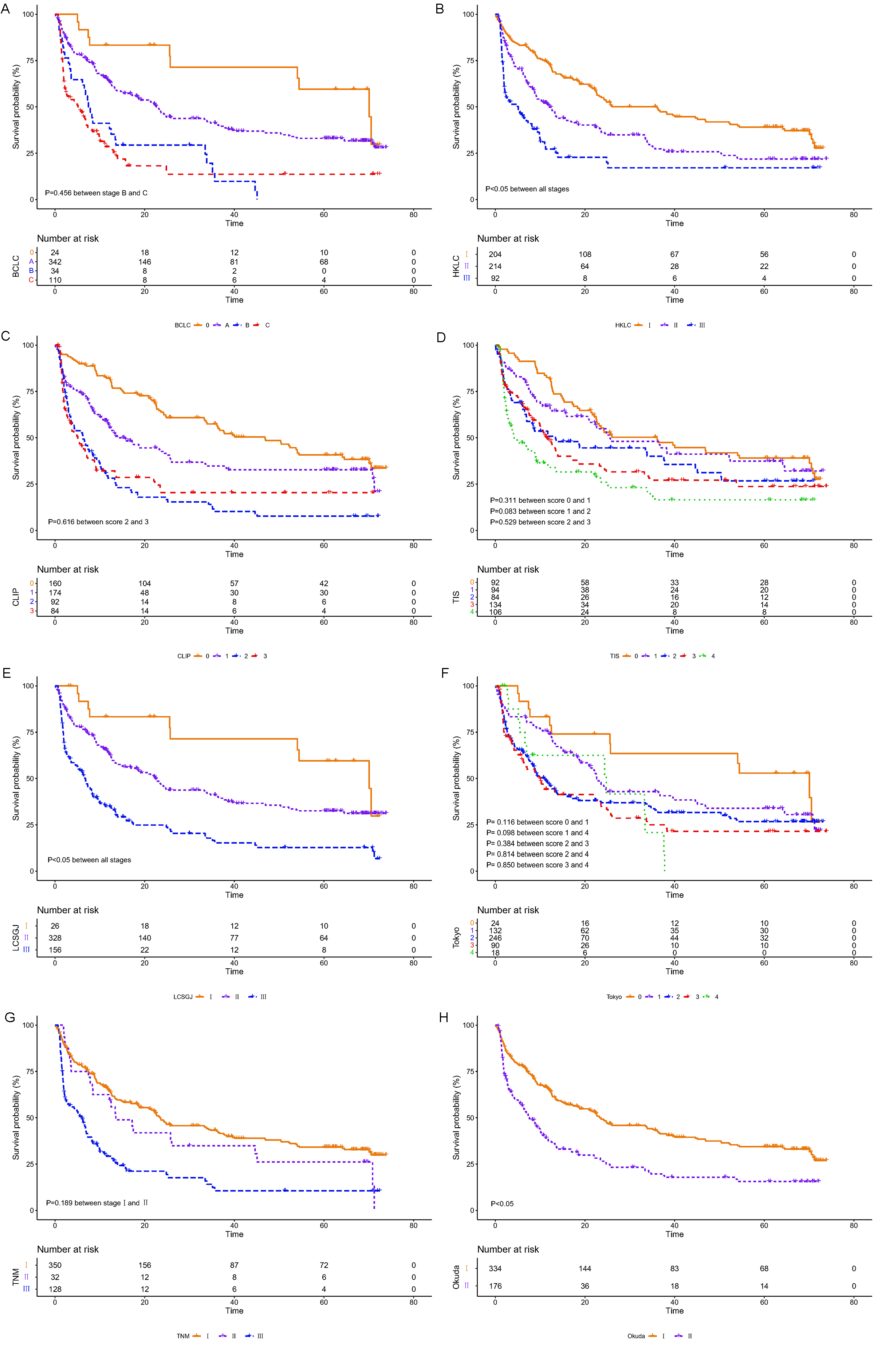

Supplement: Supplementary Figure 2 — Comparison of disease-free survival distributions by (A) Barcelona Clinic Liver Cancer, (B) Hong Kong Liver Cancer, (C) Cancer of the Liver Italian Program, (D) Taipei Integrated Scoring, (E) Tumor-Node-Metastasis by Liver Cancer Study Group of Japan, (F) Tokyo, (G) Tumor-Node-Metastasis by American Joint Cancer Committee 7th edition, and (H) Okuda staging systems in the microvascular invasion (MVI) cohort. [file Image_2.tif]

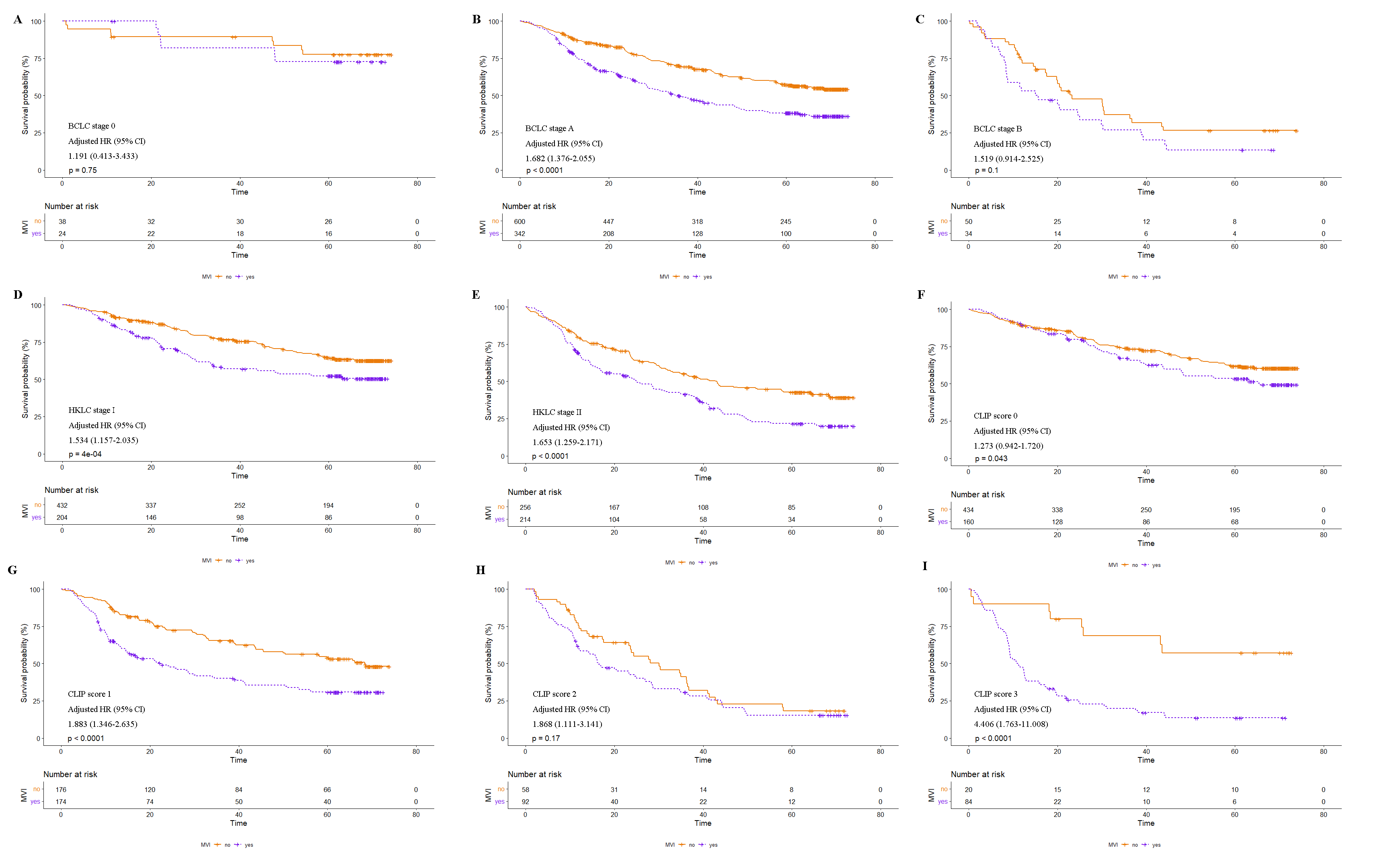

Supplement: Supplementary Figure 3 — Cumulative overall survival (OS) curves of patients with or without microvascular invasion (MVI). (A) BCLC stage 0, (B) BCLC stage A, (C) BCLC stage B, (D) HKLC stage I, (E) HKLC stage II, (F) CLIP score 0, (G) CLIP score 1, (H) CLIP score 2, (I) CLIP score 3. BCLC, Barcelona Clinic Liver Cancer; HKLC, Hong Kong Liver Cancer; CLIP, Cancer of the Liver Italian Program. [file Image_3.tif]

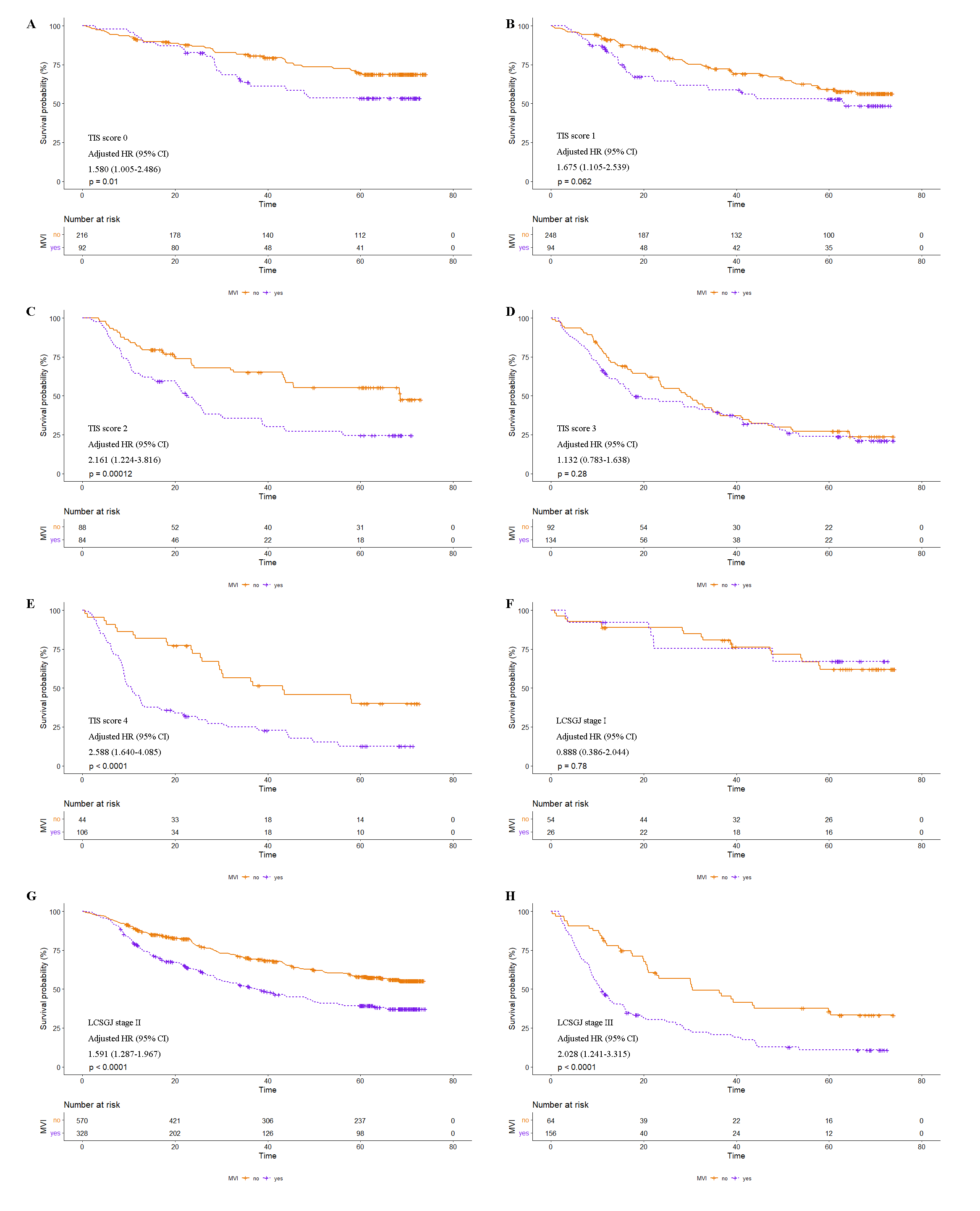

Supplement: Supplementary Figure 4 — Cumulative overall survival (OS) curves of patients with or without microvascular invasion (MVI). (A) TIS score 0, (B) TIS score 1, (C) TIS score 2, (D) TIS score 3, (E) TIS score 4, (F) LCSGJ stage I, (G) LCSGJ stage II, (H) LCSGJ stage III. TIS, Taipei Integrated Scoring System; LCSGJ, Liver Cancer Study Group of Japan. [file Image_4.tif]

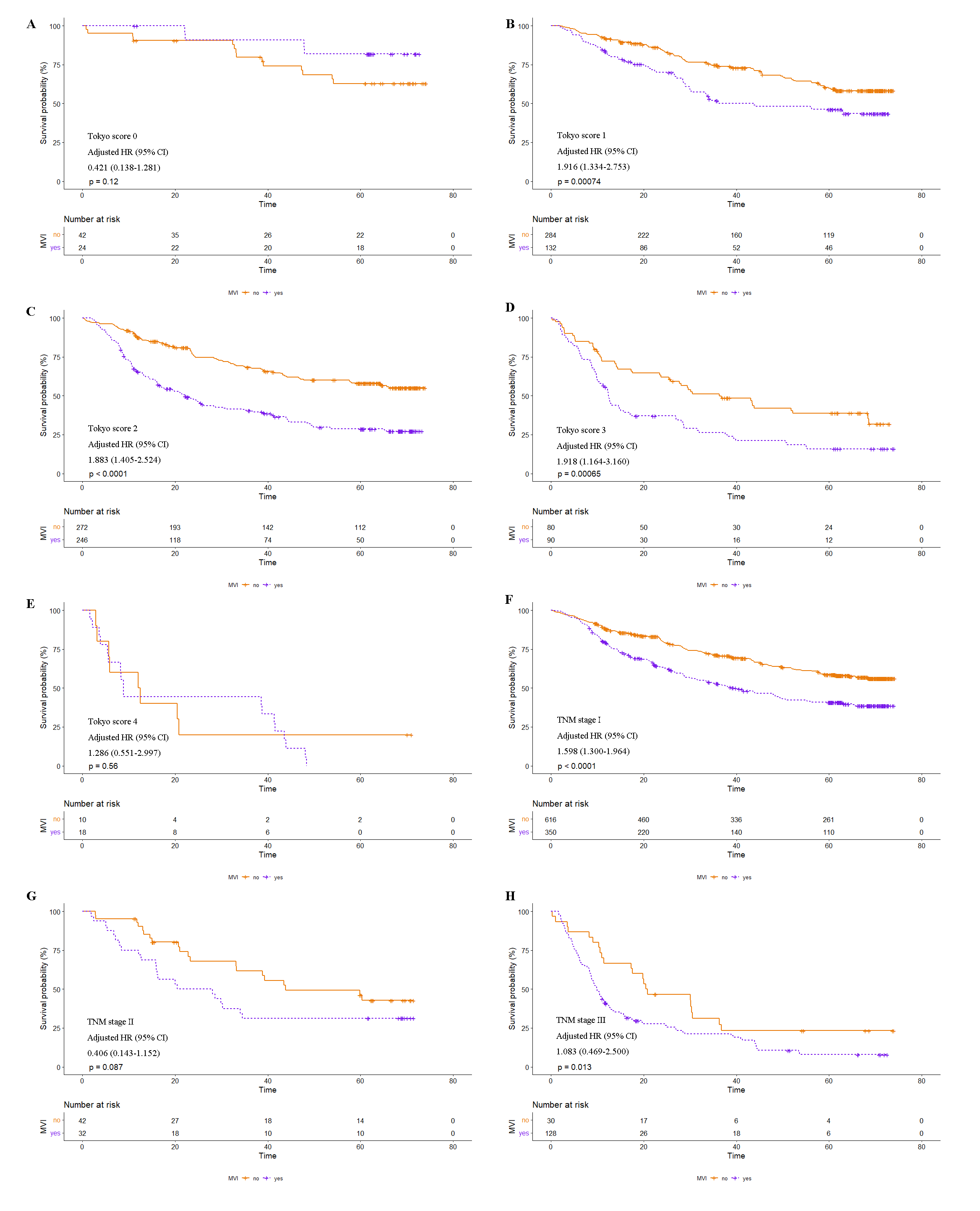

Supplement: Supplementary Figure 5 — Cumulative overall survival (OS) curves of patients with or without microvascular invasion (MVI). (A) Tokyo score 0, (B) Tokyo score 1, (C) Tokyo score 2, (D) Tokyo score 3, (E) Tokyo score 4, (F) AJCC TNM 7th stage I, (G) AJCC TNM 7th stage II, (H) AJCC TNM 7th stage III. AJCC, American Joint Cancer Committee; TNM, Tumor-Node-Metastasis. [file Image_5.tif]

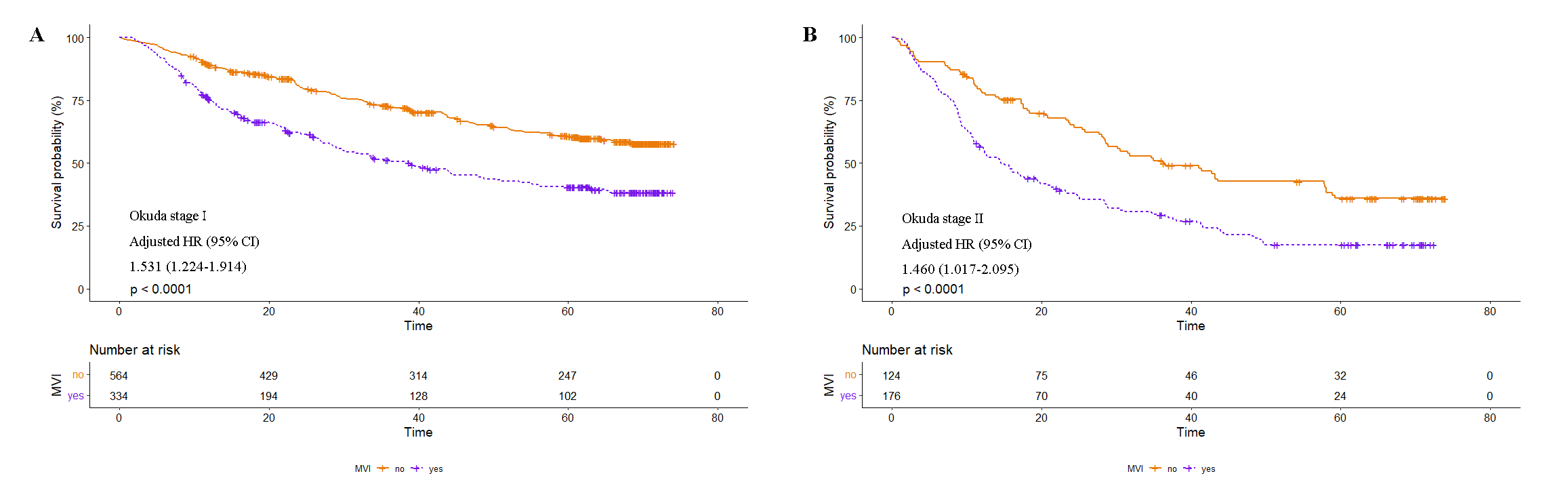

Supplement: Supplementary Figure 6 — Cumulative overall survival (OS) curves of patients with or without microvascular invasion (MVI). (A) Okuda stage I, (B) Okuda stage II. [file Image_6.tif]
