# Supplementary material for: Prognostic Value of Microvascular Invasion in Eight Existing Staging Systems for Hepatocellular Carcinoma: A Bi-Centeric Retrospective Cohort Study
Source: Front Oncol. 2021 Dec 16;11:726569. doi: 10.3389/fonc.2021.726569 (PMC8716381; doi:10.3389/fonc.2021.726569)
Supplement: Supplementary file 7 [file Table_1.docx]

Supplementary Table 1. Comparison of baseline demographics of patients with or without MVI

| Characteristics | nMVI cohort (n=688) | MVI cohort (n=510) | *P* value |
| --- | --- | --- | --- |
| Sex (male) | 540 (78.5) | 454 (89.0) | <0.001 |
| Age (≥65 years) | 92 (13.4) | 44 (8.6) | 0.013 |
| HBsAg (positive) | 546 (79.4) | 448 (87.8) | <0.001 |
| Diabetes mellitus (yes) | 66 (9.6) | 24 (4.7) | 0.002 |
| Current smoking (yes) | 234 (34.0) | 184 (36.1) | 0.463 |
| Alcoholism (yes) | 182 (26.5) | 138 (27.1) | 0.843 |
| BDTT (yes) | 4 (0.6) | 14 (2.7) | 0.003 |
| Macrovascular invasion (yes) | 0 (0.0) | 110 (21.6) | <0.001 |
| Liver cirrhosis (yes) | 458 (66.6) | 346 (67.8) | 0.664 |
| Ascites (present) | 54 (7.8) | 60 (11.8) | 0.028 |
| Hilar occlusion time (>30 min) | 36 (5.2) | 24 (4.7) | 0.789 |
| Varicose veins of gastric fundus (yes) | 96 (14.0) | 62 (12.2) | 0.388 |
| Bilirubin (≥1 mg/dl) | 174 (25.3) | 154 (30.2) | 0.067 |
| Albumin (<3.5 g/dl) | 26 (3.8) | 30 (5.9) | 0.097 |
| Prealbumin (<280 mg/L) | 556 (80.8) | 432 (84.7) | 0.091 |
| Alanine aminotransferase (>40 U/L) | 288 (41.9) | 264 (51.8) | 0.001 |
| Creatinine (≥1 mg/dl) | 8 (1.2) | 12 (2.4) | 0.17 |
| Platelet (<100 *10^9/L) | 172 (25.0) | 88 (17.3) | 0.001 |
| Alpha-fetoprotein (≥20 ng/ml) | 342 (49.7) | 354 (69.4) | <0.001 |
| Maximal tumor diameter (>5 cm) | 252 (36.6) | 288 (56.5) | <0.001 |
| Tumor nodules (multiple) | 72 (10.5) | 60 (11.8) | 0.514 |

*Abbreviations: MVI microvascular invasion; HBsAg, hepatitis B surface antigen; min, minutes; BDTT, bile duct tumor thrombus.*

*Macrovascular invasion including portal vein tumor thrombus (PVTT) and hepatic vein tumor thrombus (HVTT).*
