# Supplementary material for: Prognostic Value of Microvascular Invasion in Eight Existing Staging Systems for Hepatocellular Carcinoma: A Bi-Centeric Retrospective Cohort Study
Source: Front Oncol. 2021 Dec 16;11:726569. doi: 10.3389/fonc.2021.726569 (PMC8716381; doi:10.3389/fonc.2021.726569)
Supplement: Supplementary file 8 [file Table_2.docx]

Supplementary material

1. Barcelona Clinic Liver Cancer (BCLC) stage system

| BCLC stage | ECOG PS | Tumor status | Liver function status |
| --- | --- | --- | --- |
| 0 | 0 | Single tumor ≤ 2cm | Bilirubin was normal without portal hypertension |
| A |  |  |  |
| A1 | 0 | Single tumor | Bilirubin was normal without portal hypertension |
| A2 | 0 | Single tumor | Bilirubin was normal with portal hypertension |
| A3 | 0 | Single tumor | Bilirubin not normal and with portal hypertension |
| A4 | 0 | 3 nodules ≤ 3cm | Child-Pugh A-B |
| B | 0 | Multinodular | Child-Pugh A-B |
| C | 1-2 | Vascular invasion or metastasis | Child-Pugh A-B |
| D | 3-4 | Any | Child-Pugh C |

1. Hong Kong Liver Cancer (HKLC) stage system

| HKLC stage | ECOG PS | Child-Pugh | Tumor status | Extrahepatic vascular invasion or metastasis |
| --- | --- | --- | --- | --- |
| Ⅰ | 0 | A | Early | No |
| Ⅱa | 1 | B | Early | No |
| Ⅱb | 0-1 | A | Intermediate | No |
| Ⅲa | 0-1 | B | Intermediate | No |
| Ⅲb | 0-1 | A/B | Locally advanced | No |
| Ⅳa | 0-1 | A | Any | Yes |
| Ⅳb | 0-1 | B | Any | Yes |
| Ⅴa | 2-4 | C | Early | No |
| Ⅴb | 2-4 | C | Intermediate or Locally advanced | Yes |

Definition of Liver Tumor Status

| Liver Tumor Status | Size | Number of nodules | Intrahepatic vascular invasion |
| --- | --- | --- | --- |
| Early | ≤ 5cm | ≤ 3 | No |
| Intermediate | ≤ 5cm | ≤ 3 | Yes |
|  | ≤ 5cm | > 3 | No |
|  | > 5cm | ≤ 3 | No |
| Locally advanced | ≤ 5cm | > 3 | Yes |
|  | > 5cm | ≤ 3 | Yes |
|  | > 5cm | > 3 | Any |
|  | Diffuse | Any | Any |

1. Cancer of the Liver Italian Program (CLIP) scoring system

|  | Score | | |
| --- | --- | --- | --- |
| Variables | 0 | 1 | 2 |
| Child-Pugh stage | A | B | C |
| Tumor status | Single nodule and extent ≤50% of liver | Multinodular and extent ≤50% of liver | Massive or extent >50% of liver |
| AFP (ng/ml) | <400 | ≥400 |  |
| Portal vein thrombosis | No | Yes |  |

1. Taipei Integrated Scoring System (TIS)

|  | Score | | | |
| --- | --- | --- | --- | --- |
| Variables | 0 | 1 | 2 | 3 |
| Total tumor volume (cm^3^) | <50 | 50-250 | 250-500 | >500 |
| Child–Turcotte–Pugh | A | B | C |  |
| AFP (ng/ml) | <400 | ≥400 |  |  |

1. TNM stage by Liver Cancer Study Group of Japan (LCSGJ)

| Factors | 1. Single | 2. Size<2cm | 3. No vessel invasion |
| --- | --- | --- | --- |
| T1 | Fulfilling 3 factors |  |  |
| T2 | Fulfilling 2 factors |  |  |
| T3 | Fulfilling 1 factor |  |  |
| T4 | Fulfilling 0 factors |  |  |
| Stage Ⅰ | T1N0M0 |  |  |
| Stage Ⅱ | T2N0M0 |  |  |
| Stage Ⅲ | T3N0M0 |  |  |
| Stage Ⅳ-A | T4N0M0 or T1-T4N+M0 |  |  |
| Stage Ⅳ-B | T1-T4, N0 or N1, M+ |  |  |

1. Tokyo score

|  | Score | | |
| --- | --- | --- | --- |
| Variables | 0 | 1 | 2 |
| Albumin (g/dl) | >3.5 | 2.8-3.5 | <2.8 |
| Bilirubin (mg/dl) | <1 | 1-2 | >2 |
| Tumor size (cm) | <2 | 2-5 | >5 |
| Tumor number | ≤3 |  | >3 |

1. TNM stage by American Joint Cancer Committee (AJCC) 7^th^ edition

| T stage |  |  |  |
| --- | --- | --- | --- |
| T1 | Single nodule without vascular invasion | | |
| T2 | Single nodule with vascular invasion; Multinodular and maximum diameter ≤5cm | | |
| T3a | Multinodular and maximum diameter >5cm | | |
| T3b | Tumor involving the main branches of the portal vein or the hepatic vein | | |
| T4 | Invade adjacent organs other than the gallbladder, or penetrate the peritoneum | | |
| TNM stage | T | N | M |
| Ⅰ | T1 | N0 | M0 |
| Ⅱ | T2 | N0 | M0 |
| Ⅲ A | T3a | N0 | M0 |
| Ⅲ B | T3b | N0 | M0 |
| Ⅲ C | T4 | N0 | M0 |
| Ⅳ A | Any T | N1 | M0 |
| Ⅳ B | Any T | Any N | M1 |

1. Okuda stage system

|  | (+) | (-) |
| --- | --- | --- |
| Tumor size | >50% | <50% |
| Ascites | Yes | No |
| Albumin (g/dl) | <3 | >3 |
| Bilirubin (mg/dl) | >3 | <3 |
| Stage Ⅰ | Fulfilling 0 (+) |  |
| Stage Ⅱ | Fulfilling 1 or 2 (+) |  |
| Stage Ⅲ | Fulfilling 3 or 4 (+) |  |
